# Cuff inflation time significantly affects blood flow recorded with venous occlusion plethysmography

**DOI:** 10.1007/s00421-018-04056-8

**Published:** 2019-01-08

**Authors:** Rehan T. Junejo, Clare J. Ray, Janice M. Marshall

**Affiliations:** Institute of Clinical Sciences, College of Medical and Dental Sciences, University of Birmingham, Birmingham, UK

**Keywords:** Venous occlusion plethysmography, Cuff inflation, Hyperaemia, Blood flow

## Abstract

**Purpose:**

We tested whether the values of limb blood flow calculated with strain-gauge venous occlusion plethysmography (VOP) differ when venous occlusion is achieved by automated, or manual inflation, so providing rapid and slower inflation, respectively.

**Method:**

In 9 subjects (20–30 years), we calculated forearm blood flows (FBF) values at rest and following isometric handgrip at 70% maximum voluntary contraction (MVC) when rapid, or slower inflation was used.

**Result:**

Rapid and slower cuff inflation took 0.23 ± 0.01 (mean ± SEM) and 0.92 ± 0.02 s, respectively, reflecting the range reported in published studies. At rest, FBF calculated from the 1st cardiac cycle after rapid and slower inflation gave similar values: 10.5 ± 1.4 vs. 9.6 ± 1.3 ml dl^− 1^ min^− 1^, respectively (*P* > 0.05). However, immediately post-contraction, FBF was ~ 40% lower with slower inflation: 54.6 ± 5.1 vs. 33.8 ± 4.2 ml dl^− 1^ min^− 1^ (*P* < 0.01). The latter value was similar to that calculated over the 3rd cardiac cycle following rapid inflation: 2nd cardiac cycle: 40.5 ± 4.5; 3rd cycle: 32.6 ± 4.5 ml dl^− 1^ min^− 1^. Regression analyses of FBFs recorded at intervals post-contraction showed those calculated over the 1st, 2nd, or 3rd cardiac cycles with rapid inflation correlated well with those from the 1st cardiac cycle with manual inflation (*r* = 0.79, 0.82, 0.79; *P* < 0.01). However, only the slope for the 3rd cycle with rapid inflation vs. slower inflation was close to unity (2.07, 1.34, and 0.94, respectively).

**Conclusion:**

These findings confirm that the 1st cardiac cycle following venous occlusion should be used when calculating FBF using VOP and, but importantly, indicate that cuff inflation should be almost instantaneous; just ≥ 0.9 s leads to substantial underestimation, especially at high flows.

## Introduction

Venous occlusion plethysmography (VOP) is widely used for measuring limb blood flow: it is relatively inexpensive, non-invasive, and apparently simple to use. VOP rests on the assumption that when venous outflow is occluded, by inflating a cuff to a pressure higher than venous pressure, limb volume increases at a rate that reflects arterial inflow. The technique originally required the limb to be sealed in an air- or water-filled chamber (Greenfield et al. 1963). Subsequently, strain-gauge VOP was introduced, which allowed change in electrical resistance of mercury-filled tubing encircling the limb to be converted to change in limb volume via a small mechanical calibration (Whitney 1953). Later, electrical calibration of the strain gauge was described (Hokanson et al. 1975) and by the 1990s, and units were commercially available that allowed electrical calibration and/or automated inflation of the venous occluding cuff.

Many studies have tested the reliability and reproducibility of VOP. Since arterial inflow gradually falls following venous occlusion as the veins fill, it was recommended in an early review (Greenfield et al. 1963) and endorsed since (Joyner et al. 2001; Wilkinson and Webb 2001) that blood flow be calculated from the initial rate of volume increase, generally over the first few cardiac cycles. Forearm, or calf blood flow (FBF, CBF, respectively) estimated over 5–6 cardiac cycles with VOP correlated well with those made by Xenon inhalation (Adiseshiah et al. 1984), electromagnetic flow probe (Longhurst et al. 1974), or Doppler ultrasound measurements of velocity and diameter of the supplying artery at rest, following exercise or head-up tilt (Pallares et al. 1994; Kooijman et al. 2007). Furthermore, VOP measurements were highly reproducible at rest and during reduced or increased blood flow, more reproducible than Doppler ultrasound (Roberts et al. 1986; Pallares et al. 1994; Thijssen et al. 2005; Kooijman et al. 2007).

However, the accuracy of VOP measurements has been far more controversial. FBF or CBF measured with VOP over 5–6 s were greater following exercise than that recorded simultaneously with electromagnetic flow probe (Longhurst et al. 1974), but lower than those recorded with Doppler ultrasound (Byström et al. 1998; Pallares et al. 1994); these disparities were attributed to differences in the vascular territories measured by the techniques. By contrast, Hiatt et al. (1989) reported that CBF measured by VOP was substantially lower than that estimated by Doppler ultrasound over a comparable 6 s period without venous occlusion, at rest and following graded exercise. Since they also observed graded reductions in the diameter of the artery under the occluding cuff during inflation to 20, 40, or 60 mmHg at rest, they argued inflation attenuated limb blood flow by causing arterial collapse. This conflicted with evidence that limb blood flow measured with VOP over 5–6 s is the same at occluding pressures ranging from 30 to 60 mmHg (Wilkins and Bradley 1946; Groothuis et al. 2003).

Tschakovsky et al. (1995) gave new insight to this issue by making simultaneous, beat-to-beat measurements of FBF with VOP and brachial artery velocity with Doppler ultrasound, during breaks in rhythmic forearm contractions. Blood velocity did not decrease during the 1st cardiac cycle following inflation of the venous cuff to 50 mmHg, but fell progressively over subsequent cardiac cycles. Thus, they concluded that VOP measurements of FBF should be made over the 1st cardiac cycle following venous occlusion, so calling into question all studies in which limb blood flow was calculated over 5–6 s. Subsequently, Wood and Stewart (2010) extended these findings by measuring FBF with VOP during breaks in rhythmic contractions at 30–60% maximum voluntary contraction (MVC). Not only did FBF progressively decrease when estimates were made over the 2nd, 3rd, or 4th cardiac cycles rather than the 1st, but the disparities widened as the workload increased.

Given arterial inflow decreases as early as the 2nd cardiac cycle, a question arises as to whether the rapidity of cuff inflation affects the rate of increase in limb volume. A faster rate of occlusion would be expected to limit venous filling during occlusion, allowing the volume increase over the 1st cardiac cycle to more closely reflect the unrestricted arterial inflow. Certainly, Greenfield et al. (1963) recommended using a cuff attached to a large pressure reservoir with short, large bore tubing so as to abruptly occlude venous outflow. However, contemporaneous recordings suggest that it took ~ 1 s to achieve the occluding pressure of 50 mmHg (Whitney 1953). This is longer than the values of ~ 0.75 s and < 0.3 s quoted by Tschakovsky et al. (1995) and Wood and Stewart (2010), respectively, but shorter than the value of 2.5 s quoted by Wythe et al. (2015), all of whom used commercially available, rapid inflation systems. Furthermore, many studies, including some of those mentioned above or published recently, made no mention of how the venous occluding cuff was inflated or how long it took (e.g., Roberts et al. 1986; Hiatt et al. 1989; Carter et al. 2005; Kamijo et al. 2005; Okada et al. 2015; Cramer et al. 2017). Others, including some of ours, used manual cuff inflation (e.g., Appenzeller et al. 1963; Imms et al. 1988; Feliciani et al. 2016; Fordy and Marshall 2012; Caruana and Marshall 2015) and made no mention of inflation time.

Against this background, the aim of the present study was to compare FBF values recorded by strain-gauge VOP when using a widely available automated rapid inflation system, with a quoted inflation time of < 0.3 s and manual inflation, which our pilot studies indicated would give a slower inflation time. To give a wide range of FBF values, we recorded FBF at rest and at intervals following isometric contraction at 70% MVC, using a venous occlusion pressure of 50 mmHg. We hypothesized that rapid automated inflation would yield higher FBF values than slower, manual inflation, especially when arterial inflow is high, even if measurements were made over the 1st cardiac cycle after inflation.

## Methods

The study was approved by University of Birmingham’s Ethical Review Committee and complied with the Declaration of Helsinki.

### Subjects

Nine healthy volunteers, six men and three women, participated in the study. Their physical characteristics were age, 26.6 ± 1.0 year (mean ± SEM), height 170.8 ± 3.0 cm, and weight 73.8 ± 4.7 kg. Following review of the experimental protocol and the risks involved, each subject gave their informed consent. All refrained from consuming caffeinated drinks for 12 h and from alcohol and participation in strenuous exercise for 24 h prior to the experiment.

### Experimental setup

Experiments were performed at room temperature; the subject sat on a couch with a backrest at ~ 65° to the horizontal, legs stretched out horizontally, and both arms resting at the level of the heart. FBF recordings were made using an electrically calibrated strain-gauge plethysmograph (EC6 plethysmograph, Hokhansen, USA). An appropriate-sized indium–gallium silastic strain gauge was mounted on the widest part of the forearm; calibrated using a 2-point volume calibration system: 0 and 1% (Tschakovsky et al. 1995; Hokanson et al. 1975). A large (~ 11 cm wide), venous occlusion cuff (SC10D rapid version cuff, Hokhansen, USA), was wrapped around the upper arm ~ 5–7 cm proximal to the elbow joint and a smaller (~ 6 cm wide) sphygmomanometer cuff was wrapped around the wrist.

**Fig. 1:**
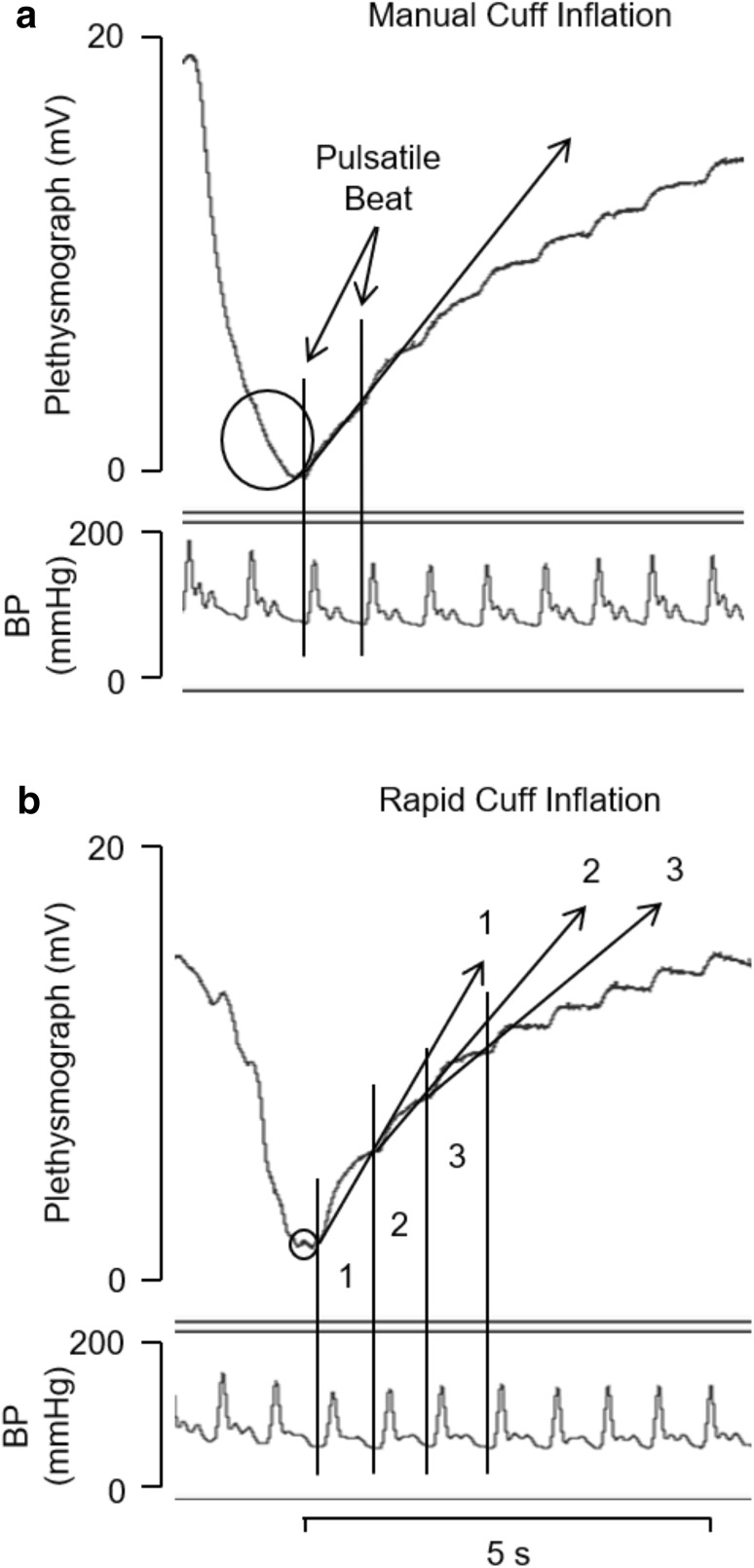
Simultaneous VOP and BP recordings immediately post-contraction following slower (**a**) and rapid inflation (**b**) of the venous occlusion cuff to 50 mmHg. The initial decrease in the plethysmograph recording indicates in each case, the change in circumference when the isometric contraction ceased. Circle on each VOP trace highlights period of time between small mechanical perturbation due to cuff inflation and beginning of increase in slope, from which period of cuff inflation was calculated. Rising arrow represents gradient over cardiac cycle/s from which FBF was calculated. Successive beat complexes marked by solid vertical lines; initial beat and gradient in **a** and 3 successive beats and their respective gradients in **b**. Pulsatile BP helps define individual cardiac cycles

For each measurement of FBF, the circulation of the hand was excluded by inflating the small cuff to ≥ 250 mmHg using a commercially available sphygmomanometer ~ 10 s before inflation of the larger cuff. The venous occlusion cuff was inflated as quickly as possible to 50 mmHg using either a standard manual sphygmomanometer (Metpak MK3, Accoson, UK), or automatically using a rapid cuff inflation system (E20 with AG101, Hokhansen, USA), slower and rapid inflation, respectively. Manual inflation was practised a few times at the beginning of each experiment to ensure occluding pressure did not overshoot 50 mmHg. FBF measurements were made offline using the initial gradient of the rising plethysmograph trace. To be specific, for both modes of inflation, the gradient was calculated over the 1st complete cardiac cycle pulse of the plethysmograph trace (Fig. 1), avoiding any initial movement artefact. Additional calculations of FBF were made using the further 2 successive cardiac cycles of rapid automated cuff inflation trace: the gradients over the 2nd and over the 3rd cycle (Fig. 1b). Meanwhile, blood pressure (BP) and heart rate (HR) were recorded from the non-exercising hand using an automatically calibrating Finapres monitor with a cuff on the middle finger (Ohmeda 2300, Finapres Medical Systems, Netherlands). All data were recorded using Power Lab bridge amplifier unit and Lab Chart data acquisition software (Version 7.3.3) at sampling frequency of 400 Hz and stored on a desktop computer.

### Protocol

Each subject reported to the lab once. Following collection of the anthropometric data, MVC was recorded by asking the subject to grip a dynamometer (Lafayette 70718, Lafayette Instruments Company, UK) with the dominant hand as hard as possible for 5 s; force recorded at the end of the 5th s was taken as MVC. The subject then rested for 10–15 min and 3 baseline FBF measurements were taken to confirm reproducibility before performing isometric handgrip contraction at 70% MVC for 1 min. An interfaced visual display unit was used, so that the subject could see and maintain the desired force; verbal encouragement was provided during the period. Post-contraction FBF was measured at 0, 15, 30 s, and 1 min and then at 1 min intervals for further 9 min. Following the last FBF measurement, the subject rested for 30 min before the protocol was repeated. The only difference between the handgrip exercise bouts was that either slower manual, or rapid automated inflation was used for venous occlusion cuff inflation. Application of the technique was randomized, but due to the nature of the experiments, subjects could not be blinded to the treatment condition.

### Analyses

All data are reported as mean ± SEM. Cardiovascular data were analysed using two-way repeated measures ANOVA for time, treatment, and interaction effects. Data were further analysed using one-way repeated measures ANOVA to detect within time effects of each individual treatment. Tukey’s HSD was used as a post-hoc test. The internal consistency of the data within and between methods was calculated as Cronbach’s alpha (*α*). Comparison of FBF values recorded with rapid automated inflation over each of the first 3 cardiac cycles and slower manual inflation, at rest and at intervals from 0 to 10 min following isometric contraction was performed by ordinary least products (OLP) regression analysis as used by Green et al. (2011) to compare measurements made with VOP and Doppler Ultrasound. This method allows for the fact that both *x* and *y* are associated with random error: slopes, *y* intercept and their associated 95% confidence intervals (CI) are reported. In addition, Pearson’s correlation coefficient (*r*) is reported to indicate the strength of the linear relationship as used by Tschakovsky et al. (1995) and Green et al. (2011). Cuff inflation times for the two venous occlusion techniques measured from the mechanical perturbations on the VOP recordings (Fig. 1), and the tension time integral (TTI) of each handgrip contraction was computed offline. These data were compared using Student’s paired *t* test. Statistical significance was assumed when *P* < 0.05.

## Results

**Fig. 2:**
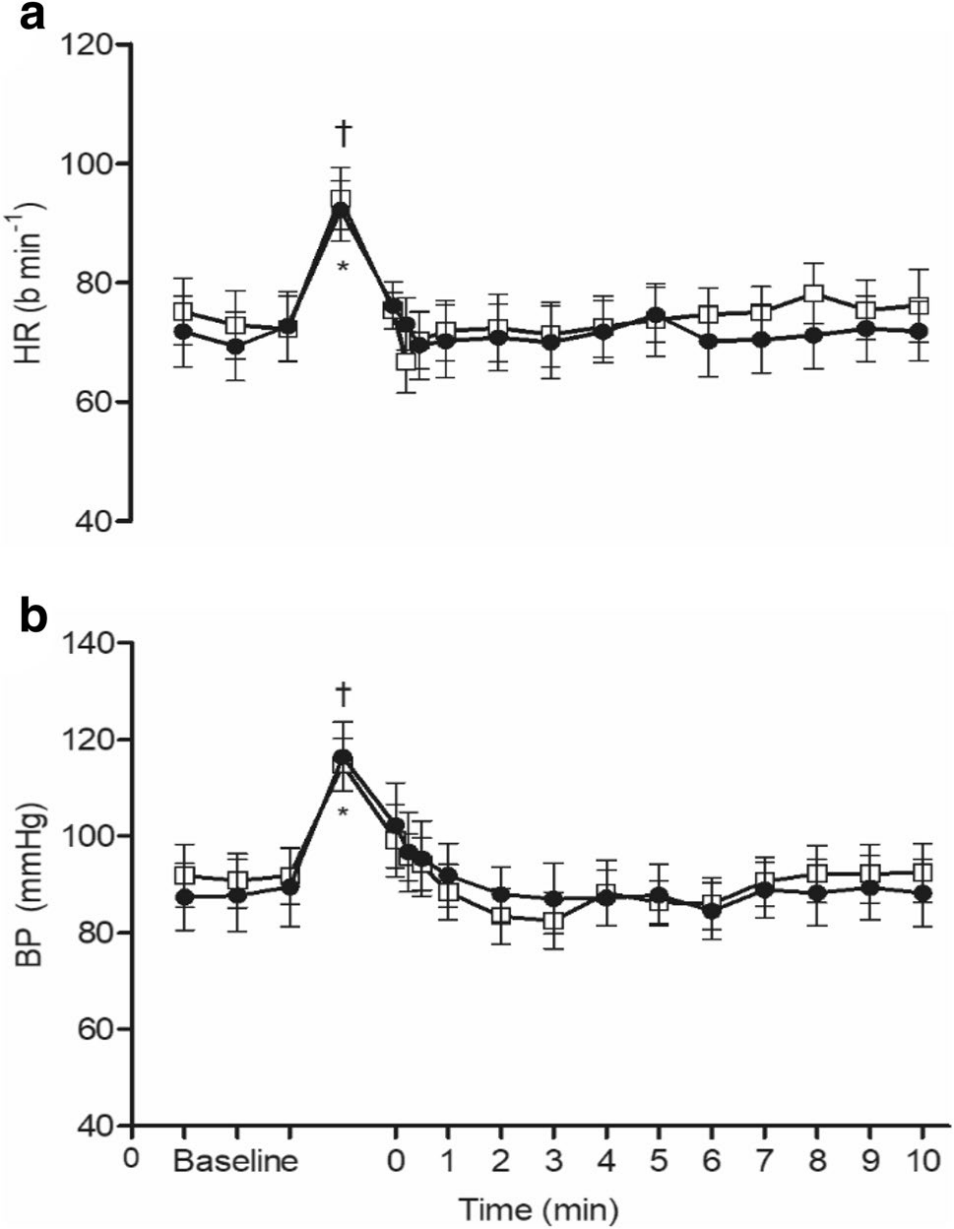
Changes in HR and BP evoked by handgrip contraction at 70% MVC for 1 min under conditions of slower manual and rapid automated inflation of venous occlusion cuff; open squares, closed circle, respectively. All values are given as mean ± SEM. *^/†^*P* < 0.05 vs. respective baselines

The TTI generated by isometric handgrip contractions during the slower manual inflation and rapid automated inflation trials was 5.1 ± 0.5 and 5.2 ± 0.6 KN s (*P* = 0.82), respectively. As shown in Fig. 2, HR (a) and BP (b) increased during isometric handgrip and returned to their respective baselines after cessation (*P* < 0.01). As expected, there was no difference between these values for the two methods of cuff inflation (*P* > 0.05).

### FBF values: slower manual inflation vs. rapid automated inflation

Time taken by slower manual inflation of the venous cuff to 50 mmHg was 0.92 ± 0.02 s. By contrast, rapid automated inflation took only 0.23 ± 0.01 s (*P* < 0.01 vs. slower manual inflation).

At baseline, i.e., under resting conditions, there was no significant difference across FBF values calculated over the 1st cardiac cycle following slower inflation and over the 1st, 2nd, and 3rd cardiac cycles following rapid inflation (Table 1). Cronbach’s *α* for the FVF values calculated by these 4 different measurements was 0.82–0.94 for the 3 baselines values, indicating good consistency between methods (Table 1). Cronbach’s *α* for FBF values calculated by each method for 3 baseline values ranged from 0.83 to 0.97, indicating good consistency within methods (Table 1).

**Table 1:** Three successive measurements of FBF (1, 2, and 3) calculated in slower inflation trial over the 1st cardiac cycle following inflation, and calculated in rapid inflation trial over the 1st, 2nd, and 3rd cardiac cycles following inflation

Baseline FBF	Slower inflation	Rapid inflation			*P* value	Cronbach’s *α*
		1st cycle	2nd cycle	3rd cycle		
1st	9.4 ± 0.9	11.1 ± 1.8	8.4 ± 1.9	8.3 ± 2.0	0.24	0.82
2nd	9.7 ± 1.2	10.2 ± 1.4	8.4 ± 1.4	7.8 ± 1.4	0.11	0.94
3rd	9.6 ± 1.3	10.5 ± 1.1	8.0 ± 1.2	7.6 ± 1.1	0.09	0.82
*P* value	0.97	0.71	0.87	0.77		
Cronbach’s *α*	0.90	0.96	0.97	0.83		

All FBF values are given as mean ± SEM in ml dl^− 1^ min^− 1^. *P* values and Cronbach’s *α* values on right-hand side indicate consistency between slower and rapid inflation, while those shown below indicate consistency between 3 baseline values within each condition

**Fig. 3:**
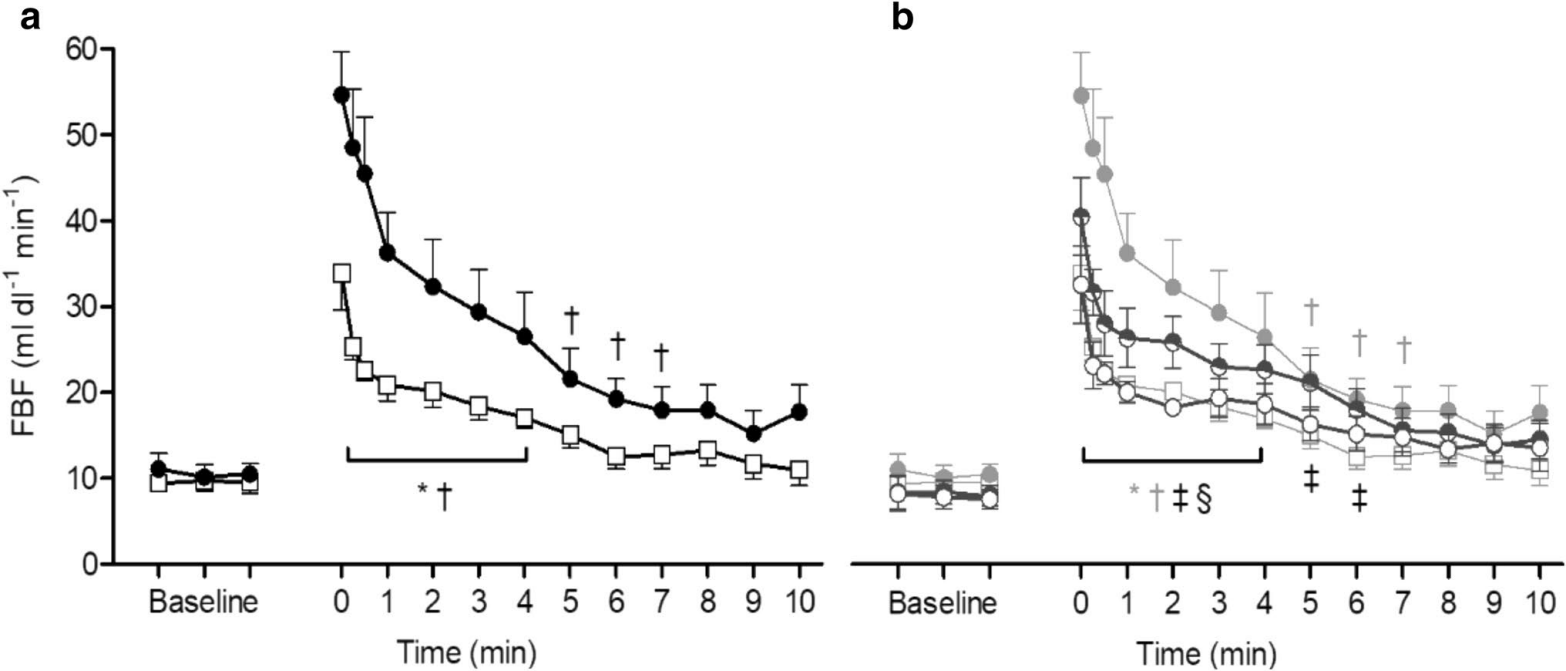
FBF values recorded before and at intervals from time 0 s to 10 min after isometric contraction at 70% MVC for 1 min, when using slower or rapid inflation. **a** FBF values recorded from 1st cardiac cycle following slower and rapid inflation (open squares, closed circles, respectively; *P* < 0.001 slower vs. rapid inflation). **b** FBF values recorded from 2nd and 3rd cardiac cycles following rapid inflation (half-closed circles and, open circles, respectively). FBF values from 1st cardiac cycle of slower and from rapid inflation as shown in **a** are repeated in grey for comparison. FBF values from 2nd and 3rd cardiac cycles were lower than those from 1st cycle of rapid inflation (*P* < 0.05). FBF values from 3rd cycle of rapid inflation were similar to those from slower inflation (*P* = 0.66). All values are given are as mean ± SEM. *^, †, ‡, §^*P* < 0.001 vs. baseline for slower cuff inflation and for 1st, 2nd, and 3rd cardiac cycles of rapid cuff inflation, respectively; bracket indicates all values within this time period

Immediately after handgrip contraction, FBF calculated with slower inflation, increased from the mean baseline value of 9.6 ± 1.2 to 33.8 ± 4.2 ml dl^− 1^ min^− 1^ (*P* < 0.001 vs. baseline); the post-contraction hyperaemia was significantly different from baseline for 4 min only (Fig. 3a). By contrast, FBF calculated from the 1st cardiac cycle following rapid inflation increased from ~ 10.5 ± 1.4 to 54.6 ± 5.1 ml dl^− 1^ min^− 1^ immediately after contraction (*P* < 0.001 vs. baseline), and remained significantly elevated for 7 min post-contraction. Comparison of post-contraction FBF values calculated with slower and rapid inflations revealed significant time (*P* < 0.001), treatment (*P* < 0.01), and interaction (*P* < 0.001) effects (Fig. 3a).

### FBF values: effect of cardiac cycles

When FBF was calculated from recordings made with rapid inflation, but using the slope over the 2nd and over the 3rd cardiac cycles, the immediate post-contraction values were 40.5 ± 4.5 and 32.6 ± 4.5 ml dl^− 1^ min^− 1^, respectively, FBF remaining elevated for 6 and 4 min post-contraction, respectively (Fig. 3b). These values were significantly lower than those calculated from the 1st cardiac cycle with rapid inflation (*P* < 0.05 and *P* < 0.01, respectively). Interestingly, the FBF values calculated using the 3rd cardiac cycle following rapid inflation were similar to those calculated from the initial gradient with slower inflation (*P* = 0.66; Fig. 3b).

### Comparisons between FBF values obtained with different rates of inflation

**Fig. 4:**
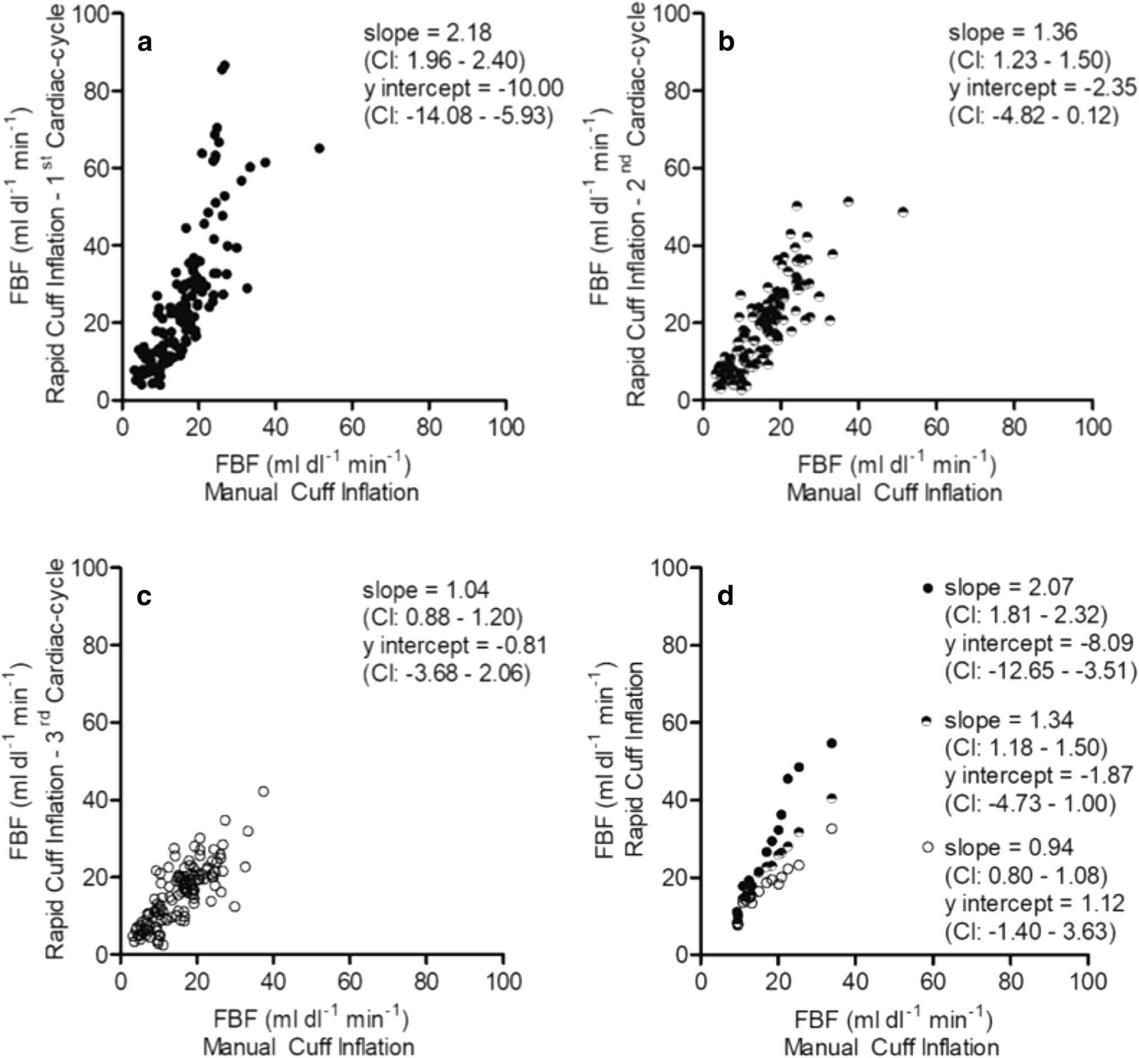
Correlations between FBF values calculated by OLP regression over 1st cardiac cycle with slower manual inflation and over 1st, 2nd, and 3rd cardiac cycles following rapid automated inflation. **a**–**c** Individual FBF values calculated at rest and at intervals following contraction; **d** mean of values calculated at each timepoint. Higher FBF values were obtained from the 1st cardiac cycle of rapid cuff inflation; by the 3rd cycle, the slope between the two inflation times was ~ 1

A high degree of correlation was observed when the FBF values calculated with slower manual inflation were compared with those calculated from the 1st, the 2nd, and the 3rd cardiac cycles with rapid automated inflation whether the values considered are the individual values at each timepoint before and after contraction (Fig. 4a–c Pearson’s *r* = 0.79, 0.82, 0.79; *P* < 0.01), or the mean values at each timepoint (Fig. 4d: Pearson’s *r* = 0.98, 0.98, 0.96; *P* < 0.01). OLP regression showed the slope between rapid and slower inflation declined progressively from the 1st to 3rd cardiac cycles whether individual values at each timepoint were considered (Fig. 4a–c), or the mean values (Fig. 4d). The values for the *y* intercept indicated a fixed bias between the measurements, and also that slower cuff inflation consistently underestimated FBF compared to rapid inflation: by 8–10 ml dl^− 1^ min^− 1^ when using the 1st cardiac cycle of rapid inflation (Fig. 4a–d). The slope was ~ 1, only between the FBF values calculated over the 1st cardiac cycle with slower inflation and over the 3rd cardiac cycle with rapid inflation (Fig. 4c, d).

## Discussion

To our knowledge, this is the first study to directly investigate the effect of time taken to inflate the venous occlusion cuff on FBF values calculated using strain-gauge VOP. In view of evidence that FBF should be calculated over the 1st cardiac cycle following cuff inflation, because FBF progressively decreases from the 2nd cardiac cycle onwards (Tschakovsky et al. 1995; Wood and Stewart 2010), our primary objective was to compare FBF values calculated from the 1st cardiac cycle following slower and rapid inflation, using manual and automated inflation, respectively, to achieve this. With slower inflation, peak FBF following isometric contraction at 70% MVC was considerably less than that calculated with rapid inflation. Furthermore, whereas FBF calculated following rapid inflation remained significantly raised until the 7th min following contraction, FBF calculated following slower inflation was greater than baseline only until the 4th min. Thus, slower inflation greatly underestimated both the peak and time course of post-contraction hyperaemia relative to rapid inflation.

The fact that it took almost four times longer to achieve the desired occluding pressure of 50 mmHg with slower manual inflation than rapid automated inflation (0.23 vs. 0.92 s), inevitably introduced a time delay before FBF could be calculated. The delay itself could not explain the discrepancy between the values obtained. For example, the difference between FBF measured at peak hyperaemia and 15 s later with rapid inflation was only ~ 12% of the peak value; far less than the 40% discrepancy between peak values calculated following slower manual and rapid automated inflation. The explanation is much more likely to lie in the additional venous filling allowed during inflation by the slower rate of inflation (see Brown et al. 1966), such that the perfusion pressure gradient was already reduced when the occluding pressure of 50 mmHg was reached.

The comparisons we made between FBF values calculated after slower manual inflation and those made over successive cardiac cycles following rapid inflation agree with that interpretation. For example, following rapid inflation, peak FBF following 70% MVC fell progressively when calculated over the 2nd and 3rd, rather than the 1st cardiac cycle, in agreement with Wood and Stewart (2010) who used the same automated inflation system as us and estimated peak FBFs at 15–60% MVC. Furthermore, following rapid inflation, FBF values calculated over the 1st, 2nd, and 3rd cardiac cycles from peak hyperaemia until return to baseline each correlated well with those calculated over the 1st cardiac cycle following slower inflation. However, the regression line was closest to unity for FBF values calculated over the 3rd cardiac cycle with rapid automated inflation and the 1st cardiac cycle with slower manual inflation. These findings can be reconciled, because slower manual inflation took almost 1 cardiac cycle at rest, but ~ 2 cardiac cycles when HR and FBF were raised following exercise, whereas rapid automated inflation took only a small portion of 1 cardiac cycle under both conditions. Thus, the 1st complete cardiac cycle over which it was possible to calculate FBF following slower inflation corresponded with the 2nd or 3rd cardiac cycle from the end of rapid inflation. Indeed, our results allow us to argue that cuff inflation time must be ≤ 0.3 s to ensure that high FBFs can be accurately calculated from the 1st cardiac cycle, even when HR increases to 180–200 beats min^− 1^.

### Wider implications

The present findings add to existing evidence that any time delay in the period over which FBF is calculated when using VOP leads to underestimation, particularly at high flows. Such delays include calculating FBF over the 2nd, 3rd, or 4th cardiac cycles rather than the 1st (Tschakovsky et al. 1995; Wood and Stewart 2010), using the cumulative slope over several cardiac cycles (Wood and Stewart 2010) and, as we now show, using cuff inflation that takes longer than one cardiac cycle. Although we compared manual and automated inflation to achieve two different rates of inflation, it is reasonable to assume our results reflect the duration, rather than the method of inflation. Furthermore, there is no obvious reason why these same principles should not apply when using VOP for measurement of CBF. Certainly, when the venous occlusion cuff was kept inflated during rhythmic calf muscle contractions at 30–70% MVC, so avoiding cuff inflation time, CBF measured over the 1st cardiac cycle during the relaxation phases, compared very favourably with values estimated by Doppler ultrasound (Green et al. 2011; Murphy et al. 2018).

These points are highly relevant, because even in some studies published since the key study of Tschakovsky et al. (1995), a 1–2 s delay was allowed after completing venous occlusion and before limb flow was calculated, and/or flow was calculated over 4–6 s (e.g., Groothuis et al. 2003; Thijssen et al. 2005; Kooijman et al. 2007). Indeed, Wythe et al. (2015) recently recommended in a methodological study on VOP that a delay as long as 4 s should be allowed before calculating FBF over a period of 2 s, i.e., at least 2 cardiac cycles. Furthermore, their statement that the same rapid inflation system as we used, with a manufacturer’s quoted inflation time of 0.3 s, took 2.5 s to inflate the occluding cuff to 50 mmHg, suggests actual inflation time may reflect factors other than the inflation system per se. As we indicated in the Introduction, periods of up to 0.75 s and < 0.3 s have been quoted for commercially available, rapid inflation systems (Tschakovsky et al. 1995; Wood and Stewart 2010), while some studies used manual inflation, and others provided no information at all on the method of cuff inflation.

In view of this variability in the application of VOP, it seems that changes in absolute FBF must have been underestimated in many published studies, especially during vasodilator stimuli such as exercise and reactive hyperaemia, mental stress, and heating. This does not devalue the physiological significance of these findings, or the contribution VOP has made to understanding of peripheral vascular regulation (see Joyner et al. 2001), and it simply questions the absolute values reported. Moreover, any underestimation of high limb flows in pharmacological studies (see Wilkinson and Webb 2001) must have made the effects of antagonists on vasodilator responses more difficult to differentiate. Looking back, it is paradoxical that in early studies with VOP, and it was recognised that the inflation system should be optimised using a cuff of small distended volume, large pressure reservoir, and short, wide-bore connexions so as to achieve venous occlusion pressure as rapidly as possible. Furthermore, it was accepted that recordings in which apparent rate of inflow falls from beat to beat, i.e., the slope from which flow is calculated, are difficult to interpret and should be analysed by joining corresponding points on pulse waves that “lie in a straight line” (Greenfield et al. 1963; Roddie and Wallace 1979). We still seem to be re-learning and exploring these issues.

Looking forward, we argue that in any new study involving VOP, information should be provided on cuff inflation time, as well as on the timing and duration of the slope used to calculate FBF, or CBF. Such information has wider application for venous occlusion is often combined with near-infrared spectroscopy (NIRS) to allow calculation of limb muscle blood flow, or oxygen consumption (VO_2_) from the rate of increase in total haemoglobin (THb), or deoxygenated haemoglobin (HHb) following venous occlusion (Homma et al. 1996; Van Beekvelt et al. 2001; Malagoni et al. 2010). Cuff inflation times of up to 4 s and slope durations of 3–80 s were used for calculation in these studies. However, recently, when Cross and Sabapathy (2017) used automated rapid cuff inflation they found that muscle blood flow calculated from rate of increase in THb decreased progressively from the 1st cardiac cycle onwards, the decrement being greater at high flows, just like FBF calculated from the rate of increase in limb volume when using VOP. Thus, it is reasonable to deduce cuff inflation time is just as important when calculating muscle blood flow or VO_2_ with NIRS, as we show for VOP.

### Study limitations

Clearly, it was impossible to simultaneously inflate the occluding cuff in two different ways in the same arm. Thus, we inevitably made comparisons between FBF values calculated with slower and rapid inflation for two different periods of isometric contraction in each subject. Although this must have contributed to the variability, it is unlikely it contributed to the marked disparity between FBF values calculated with the two methods given we randomised the order in which they were used. Our results relate to FBF values calculated when the venous occlusion cuff pressure was set at 50 mmHg; this value was chosen, because it is the widely used in studies involving venous occlusion plethysmography (Lorentsen et al. 1970; Tschakovsky et al. 1995; Wood and Stewart 2010). Had we used a lower pressure, for example, 30 or 40 mmHg, the time taken by manual inflation would have been shorter and the disparity between FBF measurements made over the 1st cardiac cycle with the two techniques would probably have been smaller. However, the principle that inflation time affects FBF values calculated at high flows would still hold. Furthermore, we only calculated FBF values before and following isometric contraction at 70% MVC, not during other stimuli. However, it is unlikely the mechanisms that cause vasodilatation affect the mechanical factors that determine the FBF values calculated with cuff inflation at different rates.

In conclusion, we have shown that when using VOP, a venous cuff inflation time of ~ 0.2, or 0.9 s, within the range used in studies over the last 70–80 years, has considerable impact on calculated absolute FBF, especially when FBF is high. It had already been established that absolute FBF decreases from the 1st cardiac cycle onwards following venous occlusion; therefore, the 1st cycle gives the most accurate assessment of FBF (Tschakovsky et al. 1995; Wood and Stewart 2010). We now show that an inflation time of just 0.9 s removes the benefit of calculating FBF over the 1st cardiac cycle, leading to FBF values equivalent to those calculated over the 3rd cardiac cycle with an inflation time of ~ 0.2 s. Taken together, these results indicate that cuff inflation time as well as the period over which FBF is calculated are of crucial importance in studies involving VOP. Without this information, the results cannot be fully evaluated.

## Abbreviations

ANOVA: Analysis of variance
BP: Blood pressure
CBF: Calf blood flow
CI: Confidence interval
FBF: Forearm blood flow
HHb: Deoxygenated haemoglobin
HR: Heart rate
MVC: Maximum voluntary contraction
NIRS: Near-infrared spectroscopy
OLP: Ordinary least products
THb: Total haemoglobin
TTI: Tension time integral
VO_2_: Oxygen consumption
VOP: Venous occlusion plethysmography

## References

[CR1] Adiseshiah M, Barber R, Szaz K (1984). Measurement of regional lower limb blood flow in normal humans by inhalation of ^133^Xe. Eur J Nucl Med.

[CR2] Appenzeller O, Davison K, Marshall J (1963). Reflex vasomotor abnormalities in the hands of migrainous subjects. J Neurol Neurosurg Psychiatry.

[CR3] Brown E, Greenfield D, Goei J, Plassaras G (1966). Filling and emptying of the low-pressure blood vessels of the human forearm. J Appl Physiol.

[CR4] Byström S, Jensen B, Jensen-Urstad M, Lindblad L, Kilbom A (1998). Ultrasound-doppler technique for monitoring blood flow in the brachial artery compared with occlusion plethysmography of the forearm. Scand J Clin Lab Invest.

[CR5] Carter JR, Kupiers NT, Ray CA (2005). Neurovascular responses to mental stress. J Physiol.

[CR6] Caruana H, Marshall JM (2015). Effects of modest hyperoxia and oral vitamin C on exercise hyperaemia and reactive hyperaemia in healthy young men. Eur J Appl Physiol.

[CR7] Cramer MN, Gagnon D, Crandall CG, Jay O (2017). Does attenuated skin blood flow lower sweat rate and the critical environmental limit for heat balance during severe heat exposure?. Exp Physiol.

[CR8] Cross TJ, Sabapathy S (2017). The impact of venous occlusion per se on forearm muscle blood flow: implications for the near-infrared spectroscopy venous occlusion technique. Clin Physiol Funct Imaging.

[CR9] Feliciani G, Peron C, La Rocca A, Scuppa MF, Malavolta A, Bianchini D, Corazza I, Zannoli R (2016). Cold pressor test using strain-gauge plethysmography. Adv Physiol Educ.

[CR10] Fordy GR, Marshall JM (2012). Breathing 40% O_2_ can attenuate post contraction hyperaemia or muscle fatigue caused by static forearm contraction, depending on timing. Exp Physiol.

[CR11] Green S, Thorp R, Reeder E, Donnelly J, Fordy G (2011). Venous occlusion plethysmography versus Doppler ultrasound in the assessment of leg blood flow during calf exercise. Eur J Appl Physiol.

[CR12] Greenfield A, Whitney R, Mowbray J (1963). Methods for the investigation of peripheral blood flow. Br Med Bull.

[CR13] Groothuis JT, van Vliet L, Kooijman M, Hopman MT (2003). Venous cuff pressures from 30 mmHg to diastolic pressure are recommended to measure arterial inflow by plethysmography. J Appl Physiol.

[CR14] Hiatt WR, Huang SY, Regensteiner JG, Micco AJ, Ishimoto G, Manco-Johnson M, Drose J, Reeves JT (1989). Venous occlusion plethysmography reduces arterial diameter and flow velocity. J Appl Physiol.

[CR15] Hokanson DE, Sumner DS, Strandness DE (1975). An electrically calibrated plethysmograph for direct measurement of limb blood flow. IEEE Trans Biomed Eng.

[CR16] Homma S, Eda H, Ogasawara S, Kagaya A (1996). Near-infrared estimation of O_2_ supply and consumption in forearm muscles working at varying intensity. J Appl Physiol.

[CR17] Imms F, Lee W-S, Ludlow P (1988). Reactive hyperaemia in the human forearm. Q J Exp Physiol.

[CR18] Joyner MJ, Dietz NM, Shepherd JT (2001). From Belfast to Mayo and beyond: the use and future of plethysmography to study blood flow in human limbs. J Appl Physiol.

[CR20] Kamijo YI, Okumoto T, Takeno Y, Okazaki K, Inaki M, Masuki S, Nose H (2005). Transient cutaneous vasodilatation and hypotension after drinking in dehydrated and exercising men. J Physiol.

[CR21] Kooijman M, Poelkens F, Rongen GA, Smits P, Hopman MT (2007). Leg blood flow measurements using venous occlusion plethysmography during head-up tilt. Clin Auton Res.

[CR22] Longhurst J, Capone RJ, Mason DT, Zelis R (1974). Comparison of blood flow measured by plethysmograph and flowmeter during steady state forearm exercise. Circulation.

[CR23] Lorentsen E, Hansteen V, Sivertssen E (1970). The influence of venous collecting pressure on measurements of calf blood flow by venous occlusion plethysmography. Angiology.

[CR24] Malagoni AM, Felisatti M, Mandini S, Mascoli F, Manfredini R, Basaglia N, Zamboni P, Manfredini F (2010). Resting muscle oxygen consumption by near-infrared spectroscopy in peripheral arterial disease: a parameter to be considered in a clinical setting?. Angiology.

[CR25] Murphy E, Rocha J, Gildea N, Green S, Egaña M (2018). Venous occlusion plethysmography vs. Doppler ultrasound in the assessment of leg blood flow kinetics during different intensities of calf exercise. Eur J Appl Physiol.

[CR26] Okada Y, Best SA, Jarvis SS, Shibata S, Parker RS, Casey BM, Levine BD, Fu Q (2015). Asian women have attenuated sympathetic activation but enhanced renal–adrenal responses during pregnancy compared to Caucasian women. J Physiol.

[CR27] Pallares L, Deane C, Baudouin S, Evans T (1994). Strain gauge plethysmography and Doppler ultrasound in the measurement of limb blood flow. Eur J Clin Invest.

[CR28] Roberts D, Tsao Y, Breckenridge A (1986). The reproducibility of limb blood flow measurements in human volunteers at rest and after exercise by using mercury-in-Silastic strain gauge plethysmography under standardized conditions. Clin Sci.

[CR29] Roddie I, Wallace W (1979). Methods for the assessment of the effects of drugs on the arterial system in man. Br J Clin Pharmacol.

[CR30] Thijssen DH, Bleeker MW, Smits P, Hopman MT (2005). Reproducibility of blood flow and post-occlusive reactive hyperaemia as measured by venous occlusion plethysmography. Clin Sci.

[CR31] Tschakovsky M, Shoemaker JK, Hughson R (1995). Beat-by-beat forearm blood flow with Doppler ultrasound and strain-gauge plethysmography. J Appl Physiol.

[CR32] Van Beekvelt MC, Colier WN, Wevers RA, Van Engelen BG (2001). Performance of near-infrared spectroscopy in measuring local O_2_ consumption and blood flow in skeletal muscle. J Appl Physiol.

[CR33] Whitney R (1953). The measurement of volume changes in human limbs. J Physiol.

[CR34] Wilkins RW, Bradley SE (1946). Changes in arterial and venous blood pressure and flow distal to a cuff inflated on the human arm. Am J Physiol Legacy Content.

[CR35] Wilkinson IB, Webb DJ (2001). Venous occlusion plethysmography in cardiovascular research: methodology and clinical applications. Br J Clin Pharmacol.

[CR36] Wood RE, Stewart IB (2010). Can venous occlusion plethysmography be used to measure high rates of arterial inflow?. Eur J Appl Physiol.

[CR37] Wythe S, Davies T, Martin D, Feelisch M, Gilbert-Kawai E (2015). Getting the most from venous occlusion plethysmography: proposed methods for the analysis of data with a rest/exercise protocol. Extrem Physiol Med.

